# Supplementary material for: Gentiopicrin-Loaded Chitosan Nanoparticles as a Topical Agent for the Treatment of Psoriasis
Source: Nanomaterials (Basel). 2024 Mar 29;14(7):610. doi: 10.3390/nano14070610 (PMC11013271; doi:10.3390/nano14070610)
Supplement: Supplementary file 1 [file nanomaterials-14-00610-s001.zip › nanomaterials-2880397-supplementary.pdf]

Gentiopicrin-Loaded Chitosan Nanoparticles as aGentiopicrin-Loaded Chitosan Nanoparticles as a Topical Agent for the Treatment of Psoriasis

Guohua Cheng [1], Xiaojie Zhang [2], Huiling Zhang [3,4], Zhixuan Feng [3,4], Jiaxiu Cai [1], Jingjing Li [1], Libo Du [2,4,*] and Ke Liu [1,*]

[1] College of Life Sciences, Sichuan University, Chengdu 610065, China
[2] Stable Key Laboratory for Structural Chemistry of Unstable and Stable Species, Institute of Chemistry, Chinese Academy of Sciences, Beijing 100190, China
[3] School of Pharmacy, Shenyang Pharmaceutical University, Shenyang 110016, China
[4] Graduate School, University of Chinese Academy of Sciences, Beijing 100053, China
* Correspondence: dulibo@iccas.ac.cn (L.D.); kliu@scu.edu.cn (K.L.); Tel.: +86-01062566306 (L.D.); +86-02885415008 (K.L.)

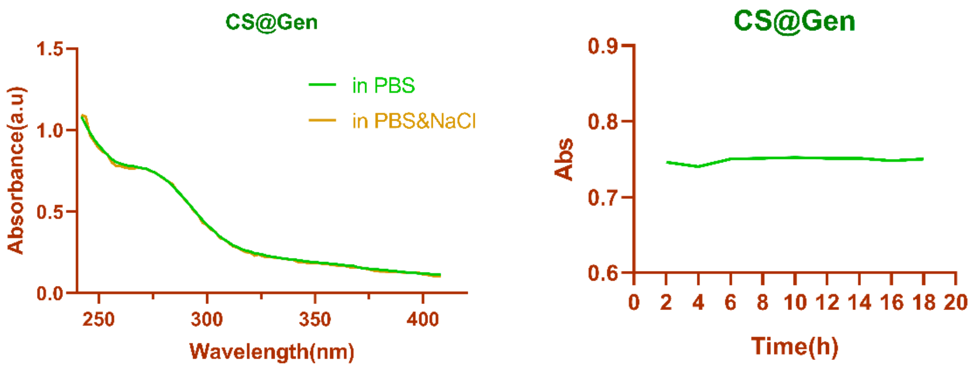

(a) (b)

Figure S1. (a) The UV-Vis spectra of CS@Gen in PBS and PBS containing NaCl; (b) The absorption intensity of CS@Gen in PBS containing NaCl.

Table S1. The size of CS@Gen after different time storage.

Time (d)	Size	Zeta Potential
3	91±4nm	7.41± 1.92 mV
7	94±7nm	7.98± 2.11 mV
15	92±6nm	8.21± 2.34 mV
30	93±6nm	8.02± 1.99 mV
60	92±6nm	8.01± 1.97 mV
90	95±7nm	8.17± 1.83 mV
